# Robot-assisted laparoendoscopic single-site surgery for the simultaneous management of multiple urinary tract calculi: a case report and experience sharing

**DOI:** 10.1186/s12894-019-0572-3

**Published:** 2019-12-30

**Authors:** Fan Zhang, Lisong Shan, Jiahui Yin, Luyang Liu, Pengchao Wang, Shengkun Sun, Xu Zhang, Hongzhao Li, Xin Ma, Gang Guo, Qiming Liu

**Affiliations:** 1 Department of Urology, The First Medical Center of Chinese PLA General Hospital, Beijing, China; 2 Department of Urology, Hainan Hospital of Chinese PLA General Hospital, Sanya, China

**Keywords:** Urolithiasis, Urinary calculi, Robotic surgery, Laparoendoscopic single-site, Case report

## Abstract

**Background:**

Urolithiasis is a clinically common benign disease in urology. Surgical treatments that are widely used in urolithiasis are percutaneous nephrolithotomy, rigid/flexible ureteroscopy, laparoscopic surgery, and endoscopic combined intrarenal surgery. The da Vinci surgical system is rarely used in the treatment of urolithiasis. In the current study, we report a case of multiple urinary tract calculi treated by robot-assisted laparoendoscopic single-site (RA-LESS) surgery.

**Case presentation:**

A 49-year-old male patient was admitted to our hospital and diagnosed with multiple urinary tract calculi. He previously underwent right ureterolithotomy, laparoscopic cholecystectomy, and extracorporeal shockwave lithotripsy. Computed tomography (CT) scan and three-dimensional reconstruction CT image showed that multiple calculi were located in the right kidney, right upper ureter, and bladder. The preoperative glomerular filtration rate (GFR) were 17.81 ml/min (right kidney) and 53.11 ml/min (left kidney). We utilized the da Vinci system docking with a single-site port to perform pyelolithotomy, ureterolithotomy, and cystolithotomy, simultaneously. The operative time was 135 min and estimated blood loss was 30 ml. The postoperative hospital stay was 5 days. Three months after surgery, the serum creatinine and urea nitrogen levels dropped to a normal range, and no residual fragments were found in the CT scan. The postoperative GFR were 26.33 ml/min (right kidney) and 55.25 ml/min (left kidney).

**Conclusions:**

RA-LESS surgery is a safe and effective surgical procedure in the treatment of multiple urinary tract calculi; however, further investigation is needed to validate its long-term therapeutic effect.

## Background

Urolithiasis is the most common urological benign disease [1], and its prevalence rate has increased worldwide in the past decades. At present, the prevalence rate of urolithiasis varies from 1 to 20% [2–4]. The common surgical procedures for multiple calculi and/or complicated calculi are percutaneous nephrolithotomy (PCNL), rigid/flexible ureteroscopy (URS), laparoscopic surgery, and endoscopic combined intrarenal surgery (ECIRS) [5–7]. However, two or more operations may be needed for patients with a heavy stone burden to achieve an ideal stone-free rate (SFR). In recent years, the surgical management of urinary tract calculi becomes increasingly efficient and minimally invasive due to new technologies and developments in equipment [8]. The da Vinci surgical system has garnered extensive interest from urologists and is widely used in tumor resection and urinary reconstruction due to its improved 3D visualization with magnification of operating field and endo-wrist technology with seven degrees of freedom that improves surgical precision [9]. Presently, robot-assisted laparoscopic surgery is rarely used in the treatment of urolithiasis. In the current study, we present a case of multiple urinary tract calculi treated by robot-assisted laparoendoscopic single-site (RA-LESS) surgery at our center.

## Case presentation

### Patient

**Fig. 1:**
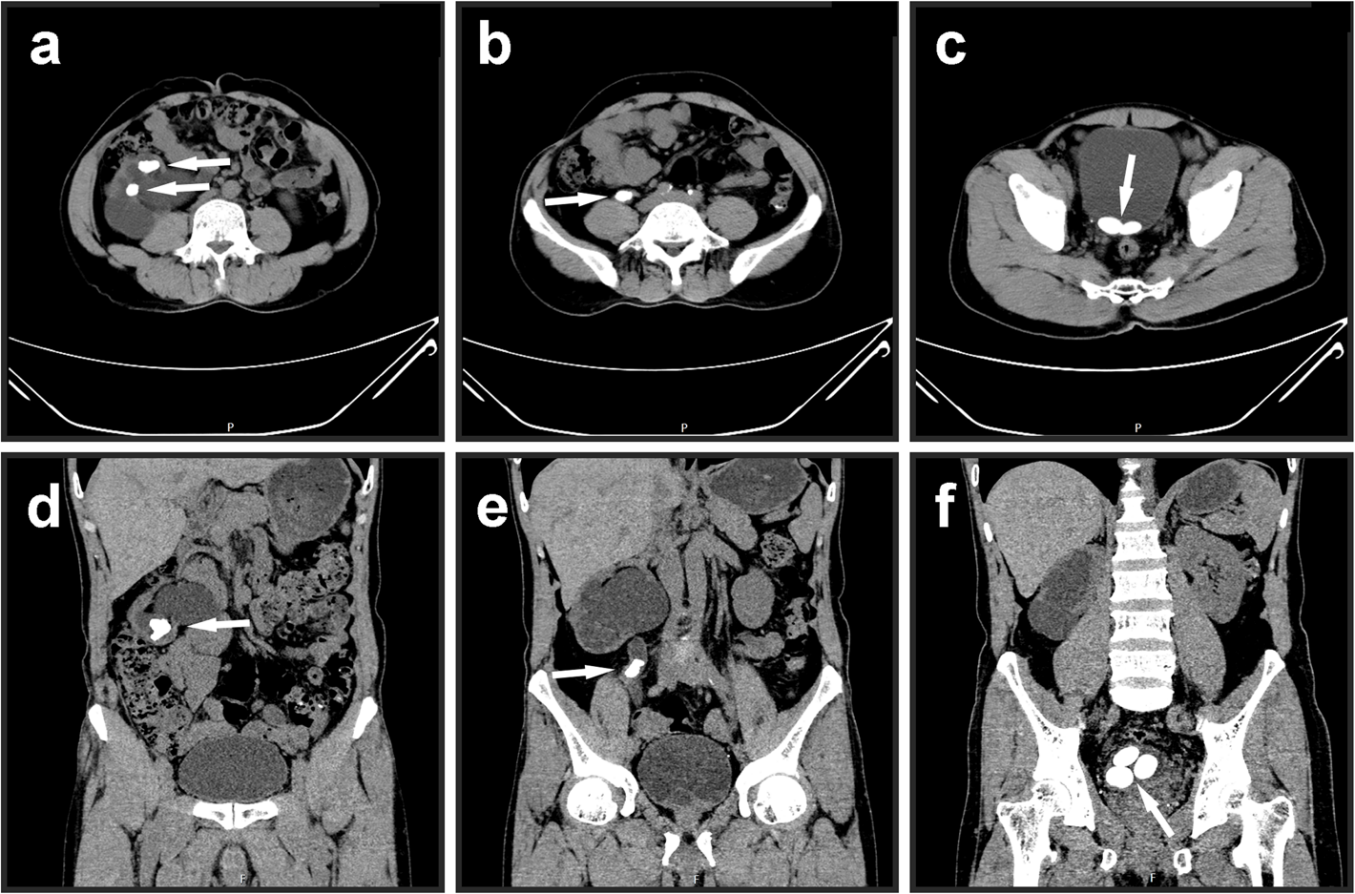
Axial and coronal images of computed tomography showing multiple urinary tract calculi (white arrows). **a** and **d** Calculi in the right kidney. **b** and **e** Calculi in the right upper ureter. **c** and **f** Calculi in the bladder

**Fig. 2:**
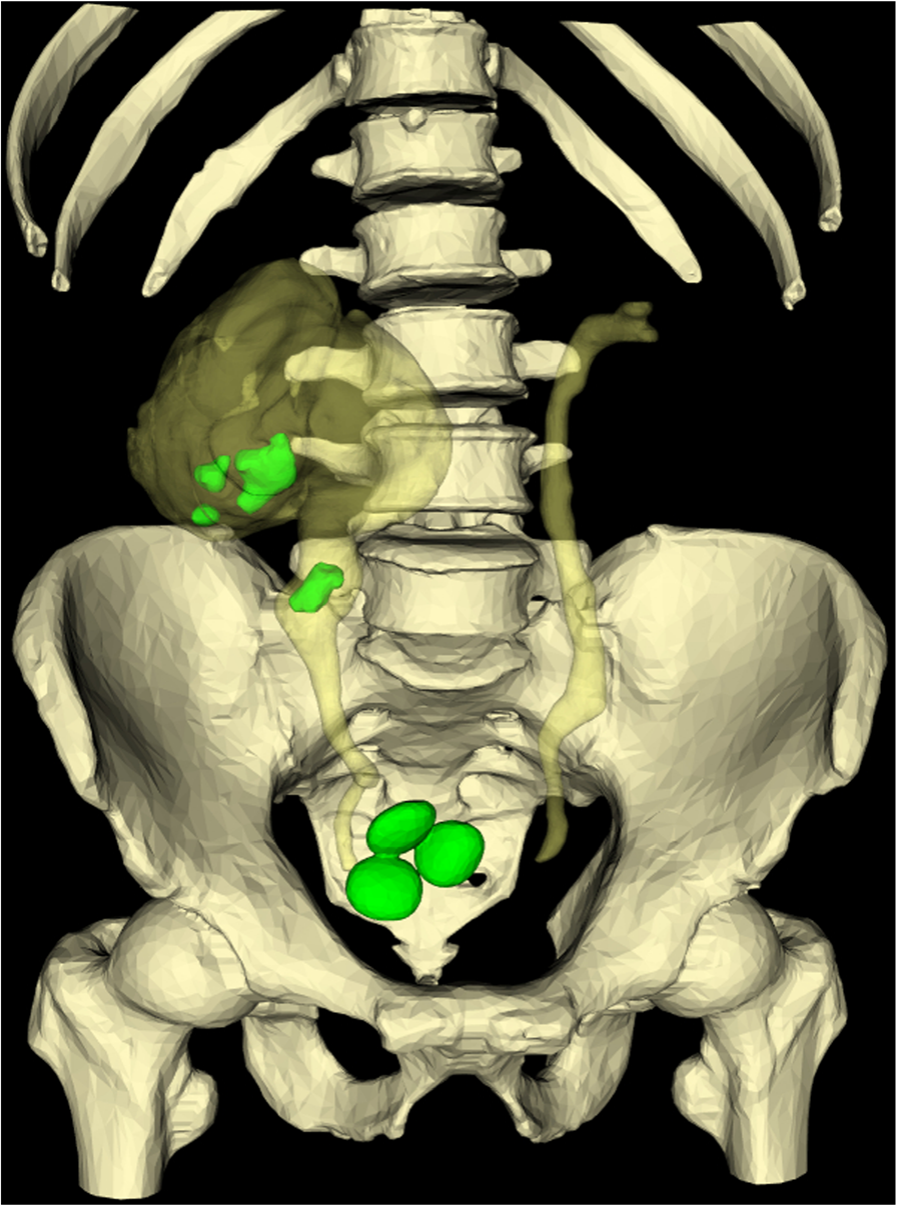
Three-dimensional reconstruction CT image showing the multiple urinary tract calculi and the morphology of the urinary tract

A 49-year-old male patient with the diagnosis of multiple urinary tract calculi at the right side was admitted to our hospital for regular operation. He had surgical histories of right ureterolithotomy in 1992 and laparoscopic cholecystectomy in 2005 and underwent extracorporeal shock wave lithotripsy (SWL) several times. No history of hypertension, heart disease, diabetes, or metabolic diseases was recorded. The body mass index (BMI) was 21.9 kg/m^2^. No other positive sign was observed during the abdominal examination except mild percussion pain in the right renal area. Laboratory tests revealed elevated serum creatinine level of 111 μmol/L (normal range: 30–110 μmol/L) and elevated urea nitrogen level of 9.3 mmol/L (normal range: 1.8–7.5 mmol/L). The result of the urine sediment analysis was within the normal range. The isotope renogram result showed that the glomerular filtration rate (GFR) were 17.81 ml/min (right kidney) and 53.11 ml/min (left kidney). Computed tomography (CT) (Fig. 1) and three-dimensional reconstruction CT image (Fig. 2) showed that three calculi were located in the right kidney, one calculi was located in the right upper ureter, and three calculi were located in the bladder. The size and hounsfield unit for the renal calculi were 3.2 cm × 1.9 cm (1174 Hu), 2.0 cm × 1.3 cm (1036 Hu), and 1.4 cm × 1.1 cm (1052 Hu), respectively (Fig. 1a and d); for the ureter calculi were 2.1 cm × 1.7 cm (1098 Hu) (Fig. 1b and e); for the bladder calculi were 2.5 cm × 1.6 cm (529 Hu), 2.3 cm × 1.5 cm (558 Hu), and 2.1 cm × 1.3 cm (564 Hu), respectively (Fig. 1c and f).

### Position and port placement

**Fig. 3:**
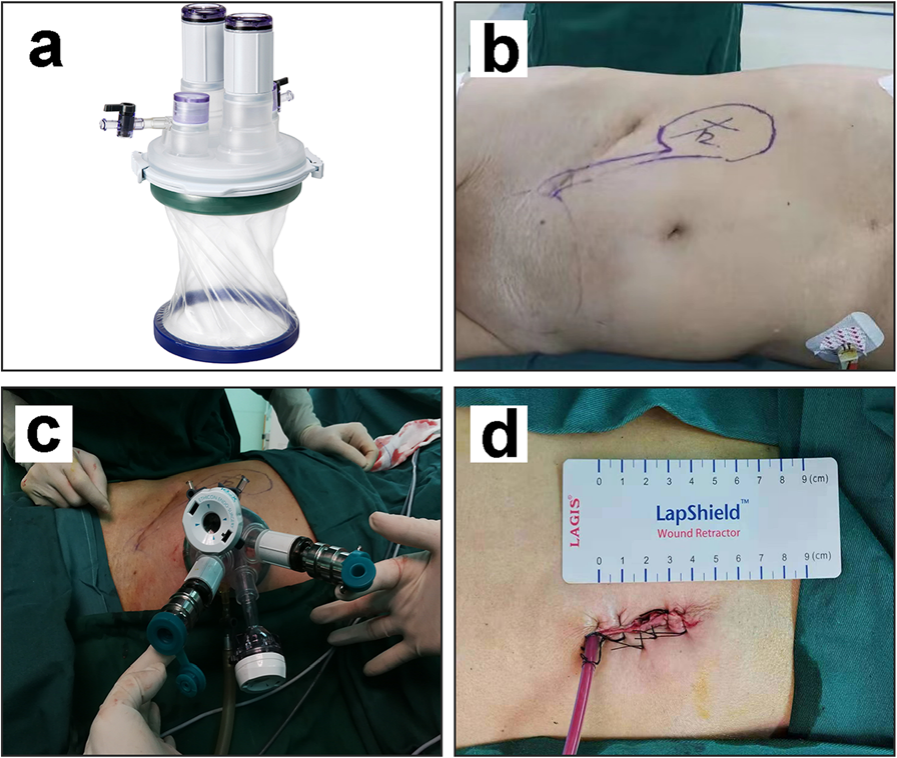
The patient position and port placement for robot-assisted laparoendoscopic single-site surgery. **a** Single-site port used for docking with da Vinci Si system. **b** Modified left lateral decubitus position with a 60°–70° bump. **c** Two 12 mm trocars are placed on the upper and lower symmetrical position, and two 8 mm trocars are placed on the left and right symmetrical position. **d** The abdominal incision after operation

The whole operation was performed through a transperitoneal approach by using the da Vinci Si system (INTUITIVE SURGICAL) and LAGIPORT™ single-site port (LAGIS ENTERPRISE CO.) (Fig. 3a). After endotracheal intubation under general anesthesia, the patient was first placed in a modified left lateral decubitus position with a 60°–70° bump (Fig. 3b). A 4 cm longitudinal incision was made around the umbilicus, and the single-site port was placed to establish the abdominal operative access. On the upper and lower symmetrical position of the port, two 12 mm trocars were placed for robot camera and assistant. On the left and right symmetrical position, two 8 mm robotic trocars were placed for the first and second robot arm (Fig. 3c). After taking out the calculi of the kidney and upper ureter, the patient was adjusted to a Trendelenburg position. The same port was used for docking robot to deal with bladder calculi. No additional incision was added during the whole procedure.

### Surgical procedure

**Fig. 4:**
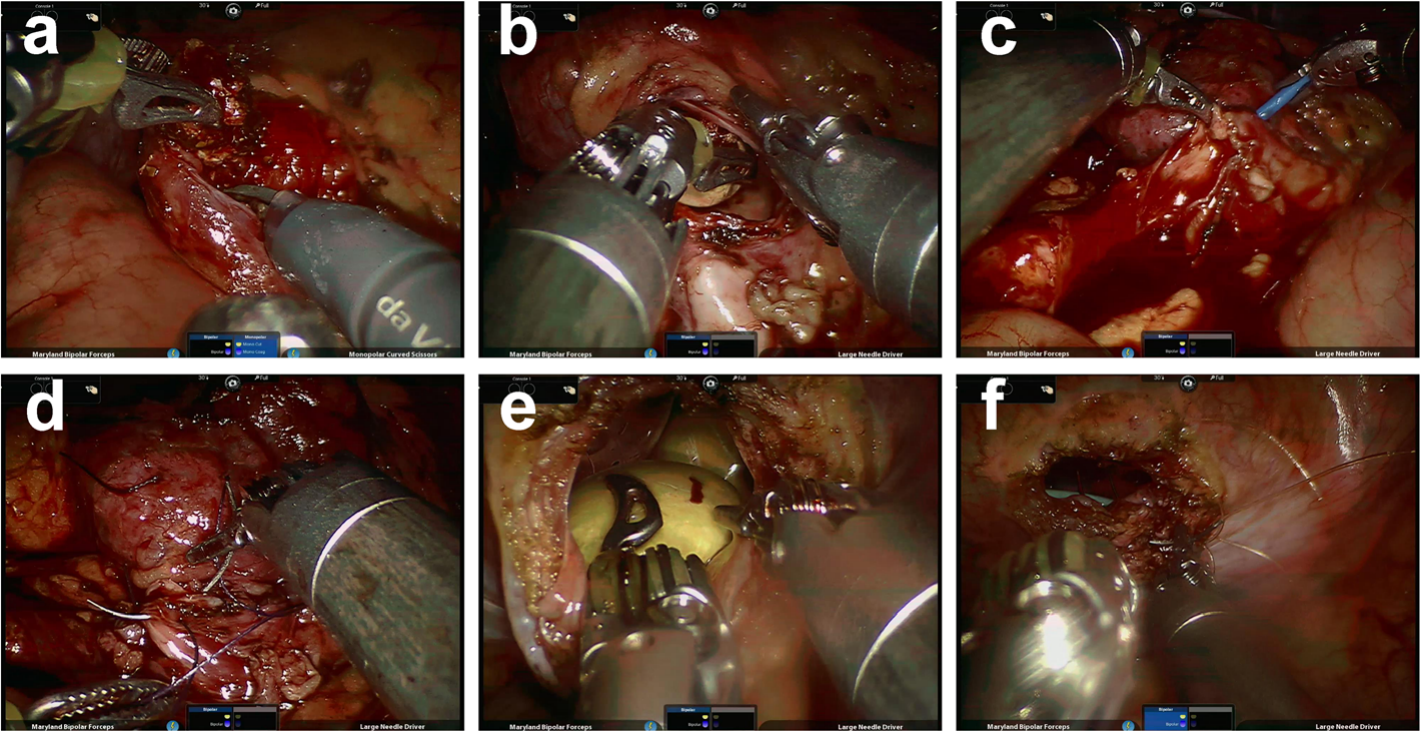
Intraoperative images of the surgical procedure. **a** The ureter calculi is removed by performing ureterolithotomy. **b** The renal calculi are removed by performing pyelolithotomy. **c** A double-J stent is indwelled into the ureter. **d** The incisions on the ureter and renal pelvis are closed with 4–0 absorbable suture. **e** The bladder calculi are removed by performing cystolithotomy. **f** The bladder incision is closed continuously with Quill™ self-retaining system absorbable surgical suture


After entering the abdominal cavity, the hepatocolic ligament was incised, and the lateral peritoneum was incised along the paracolic sulcus. Next, the Gerota’s fascia was incised to expose the right renal pelvis. The right upper ureter was tortuous seriously due to the previous ureterolithotomy related tissue adhesion. The upper ureter, was firstly isolated from the adhesion tissue, and then was dissociated carefully to expose the calculi location. Next, the ureterolithotomy and pyelolithotomy were performed according to the standard procedures (Fig. 4a and Fig. 4b). After the ureter calculi and renal calculi were all removed, the double-J stent was indwelled into the ureter (Fig. 4c), and the incisions on the ureter and renal pelvis were closed with a 4–0 absorbable suture (Fig. 4d). The Trendelenburg position was adjusted, and a Foley catheter was inserted. Then, the robot was docked with the same single-site port to perform the cystolithotomy. After the bladder calculi were removed (Fig. 4e), the incision on the bladder was closed with a Quill™ self-retaining system (SRS) absorbable surgical suture (Fig. 4f). A drainage tube was placed, and the abdominal incision was sutured after no leakage of the bladder was confirmed. An additional video file shows the surgical procedure with more details (see Additional file 1: Video clip).

**Additional file 1.** Video Clip. A short video clip of the surgical procedure.


## Results

**Fig. 5:**
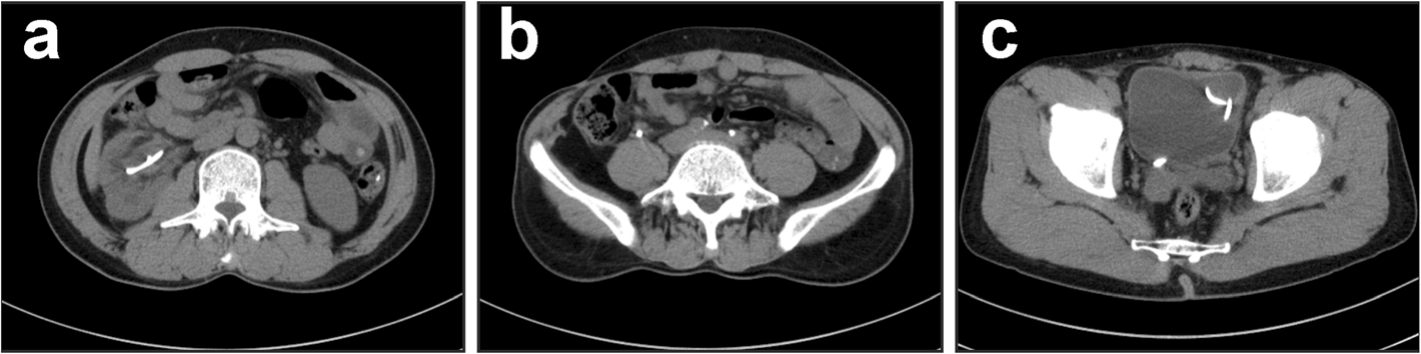
The computed tomography scan is performed 3 months after surgery. **a**, **b**, and **c** Axial images showing that no residual fragments are found

All the urinary tract calculi were totally removed by the procedure. No blood transfusion was required, and no intraoperative complication occurred. The length of the abdominal incision was 4 cm (Fig. 3d), the operative time was 135 min, and the estimated blood loss was 30 ml. The patient started off-bed activity 1 day after surgery. Three days after surgery, the patient had smooth postoperative passage of anal gas and was prescribed an oral diet. Four days after surgery, the drainage tube was removed. The total postoperative hospital stay was 5 days. No perioperative complications, such as fever, urinary leakage, bleeding, and hematuria were observed. The initial follow-up was performed 3 months after surgery. No residual fragments were found in the CT scan (Fig. 5a to c). The double-J stent was removed after finishing the CT scan, and no calculi was attached on the stent wall. The laboratory test revealed that the serum creatinine (86 μmol/L) and urea nitrogen (3.6 mmol/L) levels dropped to normal ranges. The isotope renogram result showed that the postoperative GFR were 26.33 ml/min (right kidney) and 55.25 ml/min (left kidney).

## Discussion and conclusions

Robotic surgery has brought the surgical operation third revolutionary change [10], along with laparoscopic surgery, which changed the concept of surgery. From the earliest AESOP and ZEUS to the latest da Vinci Xi, robot-assisted surgeries have been increasingly used in clinical practice. In the field of urology, da Vinci system offer advantages to tumor resection and urinary reconstruction [11, 12]. However, in the treatment of urinary calculi, da Vinci system is often used in performing nephrolithotomy [13, 14] or ureteroscopy lithotripsy during the robot-assisted pyeloplasty for ureteropelvic junction obstruction [15, 16]. No report of simultaneous treatment of calculi in the kidney, ureter, and bladder by using the da Vinci system has been found.

ECIRS is a combination of traditional PCNL and retrograde ureteroscopy. It was initially used in the year of 2008 [5], and became more and more popular in recent years. ECIRS can be utilized to remove kidney and ureter calculi simultaneously. It has been demonstrated that ECIRS is feasible and safe for patients with large and/or complex urolithiasis [6].

For the present patient, both ECIRS and RA-LESS surgery can be used as surgical methods. We finally chose RA-LESS surgery mainly based on the following considerations: first, the patient had an open ureterolithotomy history, the condition of the ureteral cavity was unclear. The preoperative CT scan showed a tortuous upper ureter, and the retrograde ureteroscopy may not be succeed in approaching the calculi location. Second, the calculi were distributed in the different renal calices, more than one working channel may be needed during the ECIRS. The potential bleeding risk may be increased during the operation. Third, pure endoscopic surgery may extend operative time and raise the chance of infection and potential perioperative complication and may thus prolong hospital stay. Lastly, the previous open ureterolithotomy may add risks and difficulties for traditional surgical methods such as ECIRS and laparoscopic surgery. Robotic surgery has more advantages in suturing and dissociating, and may avoid these unfavorable factors. We selected RA-LESS surgery instead of the traditional robot-assisted laparoendoscopic surgery in dealing with multiple urinary tract calculi because by docking with robot parked in different positions, a single-site port can be used in handling all the calculi without additional trocar, while the traditional surgery needs more than four trocars.

We found that RA-LESS surgery offers the following advantages to the treatment of multiple urinary tract calculi. First, by adjusting the robot position to dock with the same single-site port, calculi in the upper and lower urinary tracts can be removed in a single surgery. No additional trocar is required. This feature is suitable for patients with unknown ureteral conditions or urinary tract surgery histories. Second, a transperitoneal approach provides surgeons with extensive working space and prevents operative complications, such as bleeding, ureteral injuries, and infection, which are caused by PCNL and URS. Lastly, RA-LESS surgery provides surgeons with a clearer surgical field of view and more stable controllability and comfortable feeling during the operation than endoscopic or laparoscopic surgery. In addition, RA-LESS surgery does not extend hospital stay.

Meanwhile, certain disadvantages were found. First, only two robot arms can be used in the operation; the third arm cannot fully exert its advantages in exposing surgical sites and grabbing tissues. Second, with the use of single-site port and conventional da Vinci instruments, external collision of robot arms and internal collision of instruments occurred more frequently than we expected. Lastly, the indications were relatively narrow, and the cost of a single operation is expensive compared with that of endoscopic surgery.

In summary, RA-LESS surgery is safe and effective for patients with multiple urinary tract calculi and shows certain advantages for selected patients. In spite of these advantages, RA-LESS surgery only provides surgeons with another option and still cannot replace the traditional surgical methods. Furthermore, more technical details need to be optimized in the future. The long-term therapeutic effect of RA-LESS surgery requires further investigation due to the relatively few patient and short follow-up time.

## Data Availability

The datasets used and/or analyzed during the current study are available from the corresponding author on reasonable request.

## Abbreviations

BMI: Body mass index
CT: Computed tomography
ECIRS: Endoscopic combined intrarenal surgery
GFR: Glomerular filtration rate
PCNL: Percutaneous nephrolithotomy
RA-LESS: Robot-assisted laparoendoscopic single-site
SFR: Stone-free rate
SRS: Self-retaining system
SWL: Shock wave lithotripsy
URS: Ureteroscopy
